# The growth simulation of pine-needle like structure with diffusion-limited aggregation and oriented attachment†

**DOI:** 10.1039/d2ra03649e

**Published:** 2022-08-15

**Authors:** Zhijun Xia

**Affiliations:** College of Chemical and Biological Engineering, Zhejiang University Hangzhou 310028 Zhejiang Province P. R. China xiazj919@163.com; School of Pharmaceutical and Materials Engineering, Taizhou University Taizhou 318000 Zhejiang Province P. R. China

## Abstract

A growth model combined with diffusion-limited aggregation (DLA) and oriented attachment (OA) is developed for deducing quantitative understanding of the growth process of pine-needle like structures. We define the completely random parameters for describing the realistic Brownian motion in DLA. The results indicate that the cluster by DLA changes from random branches to regular needles by the introduction of OA. And the cluster of DLA and OA has a fractal dimensionality of about 1.0 during the whole growth process. The maximum length of needels (*L*_max_) depends on the number of particles (*N*_p_). They satisfy the relation *L*_max_ = *aN*_p_^*b*^ (*a* and *b* are constant) over the whole range. The model has also been used to describe the formation of needles on a line, plane and sphere. The growth of needles has obvious steric hindrance from the outer needles. In particular, only one needle grows in the later period in the plane.

## Abstract 2

A growth model combined with diffusion-limited aggregation and oriented attachment is developed for deducing the growth process of pine-needle like structure.

## Graphical abstract

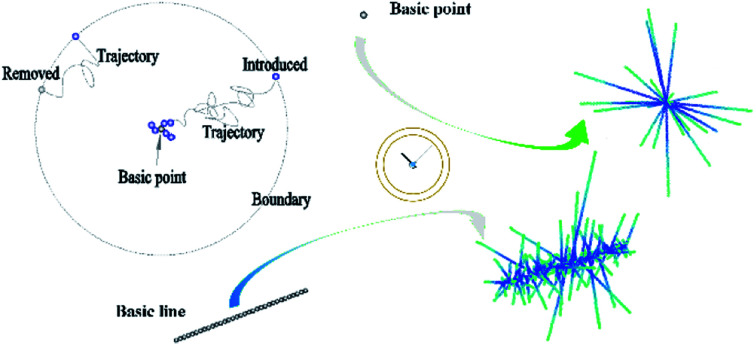

## 1. Introduction

In chemistry and materials science, the similarity of many patterns formed in non-equilibrium growth processes is conspicuous. Many modeling methods have been made to discover common underlying mechanisms. The Diffusion-Limited Aggregation (DLA) model has been used properly to deduce the dynamic growth processes arising in electro-deposition,^1–3^ dielectric breakdown,^4,5^ thin-films,^6–9^ and dendritic solidification.^10–12^ It describes random aggregation of free particles undergoing Brownian motion until they stick irreversibly to the previously formed aggregate.^13–15^ Similar non-equilibrium growth processes to DLA occur in the formation of some nanostructures, such as nanoflowers^16–20^ and pine-needles.^21–24^ However, these processes have clear growth direction due to crystallization. This is significantly different from the random aggregation.

For the numerical simulations of directed growth, the directed Diffusion-Limited-Aggregation (DDLA) model has already been considered by researchers.^25–28^ A strong external field, such as gravity or electric field, was introduced for the preferred direction of aggregation. The particles random walks in the limited direction in the field. However, due to the high complexity of particle movement and stick in the nature system, the application of these models is strictly limited. Specifically in the crystallization, the growth direction of crystal depends on itself crystalline structure and thermal gradient, rather than the field.^29–31^ For the growth of various crystals, Oriented Attachment (OA) involves spontaneous self-assembly of adjacent particles and directed aggregation, and has become a well-recognized mechanism.^31–35^

As a novel morphology, pine-needle like micro/nano-structures is attractive prospect in various fields, such as solar cells,^36,37^ capacitors,^38–40^ catalysts^41–43^ and sensors.^44–46^ The formation of this 3D nano-architectures present likely spontaneously and radially growth from bases or templates including point,^47,48^ line,^49,50^ plane^51–53^ and sphere.^54–57^ This growth process could be considered an analogous aggregation combined with random movement and OA.

In this paper, in order to simulate the growth process of pine-needle like structure, we combined the original DLA and OA mechanisms. Moreover, to simplify the model, an irreversible attachment was taken up, and neglected the influence of dynamic equilibrium and energy transfer.

## 2. Simulation method

### 2.1 Method for random motion

In our simulations, the randon numbers (Rand_N) are in the range 0–1, and generated with linear congruential method. For the randomness of Brownian motion, the motion parameters are all determined by randon numbers at each step, such as movement radius (*r* = *r*_max_ × Rand*_*1, *r*_max_ is the max free path of cluster) and motion direction (Azimuth *θ* = 2π × Rand*_*2, Vertical *ϕ* = 2π × *Rand_*3). This is different from that with specific motion parameters of the early DLA model.^14,58–60^ At every step, the displaced coordinate (*x*_*n*_, *y*_*n*_, *z*_*n*_) is calculated with the previous place (*x*_*n*−1_, *y*_*n*−1_, *z*_*n*−1_) using the equations below:^70^

1 *x_n_* = *x*_*n*−1_ + *r* cos *θ* cos *ϕ*

2 *y_n_* = *y*_*n*−1_ + *r* sin *θ* cos *ϕ*

3 *z_n_* = *z*_*n*−1_ + *r* sin *ϕ*

Where *r*, *θ* and *ϕ* are randomly at each step unless specific instruction. These equations are as well as suitable for Brownian motion of one particle in 2D plane at *ϕ* = 0 and *z* = 0.

### 2.2 DLA and OA model

**Fig. 1:**
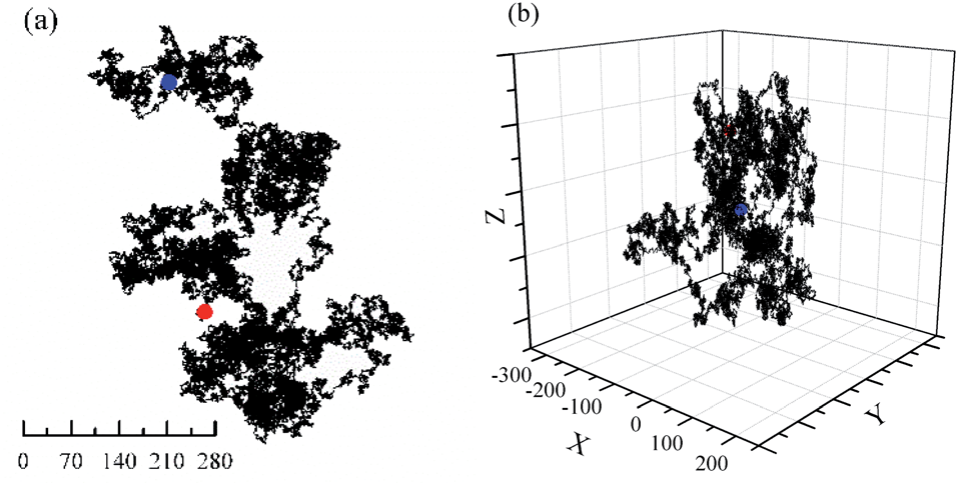
Brownian motion of one particle with 1 000 000 steps. *r*_max_ = 1, Starting point (0, 0, 0). (a) 2D plane; (b) 3D space. Red: Start; Blue: End.

**Fig. 2:**
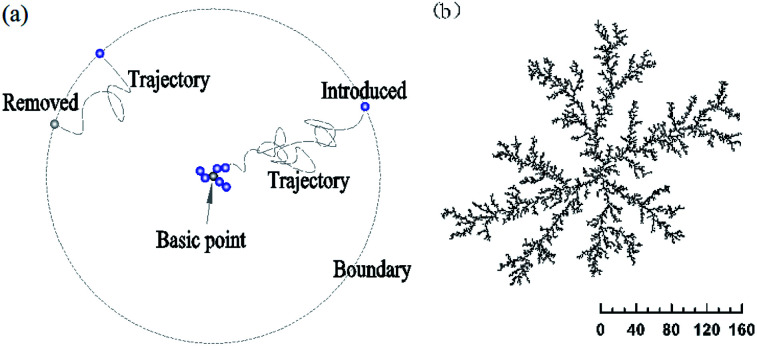
Random aggregate of DLA on a 2D plane with a basic point (0, 0). *r*_max_ = 0.5, Dist < 1, *r* and *θ* randomly, *ϕ* = 0. (a) Simulation model; (b) 2D cluster of 10 000 particles.

Our models are variant of DLA model,^13–15^ and based on the above-mentioned Brownian motion, as shown in Fig. 2a. The initial state is a seed particle at the point of origin (0, 0, 0), and as a fixed basic point. A second particle is introduced randomly at the boundary with a great distantance from the basic point (about *r*_c-max_+50 lattice units, *r*_c-max_ is the maximum radius of cluster). It walks randomly until it joins the growing cluster or gets out the boundary. Meanwhile, the next particle is added. The other particles are individually introduced and follow the second. Once the movable particle hits the cluster when their distance (Dist) is less than a constant value, it sticks irreversibly to the cluster. A detailed logic diagram of simulation process has been presented in Fig. 1s.†

For the OA, the direction of sticking is consistent with the aggregated orientation of its nearest-neighbor particle which is at the end of the outer “arms” of the growing cluster.

## 3. Results and discussion

### 3.1 Brownian motion

Computer simulations of Brownian motion for one particle in two- (2D) and three-dimensional (3D) space presents in Fig. 1. Furthermore, a more detail evolutionary process of Brownian motion in 2D plane is presented in Fig. 2s.† Their trajectorys all perform flocculent and disorganized, due to irregular motion. This result is almost the same as with specific motion parameters. However, the random walk of particle is closer to the real Brownian motion.

### 3.2 Random aggregate on 3D space

**Fig. 3:**
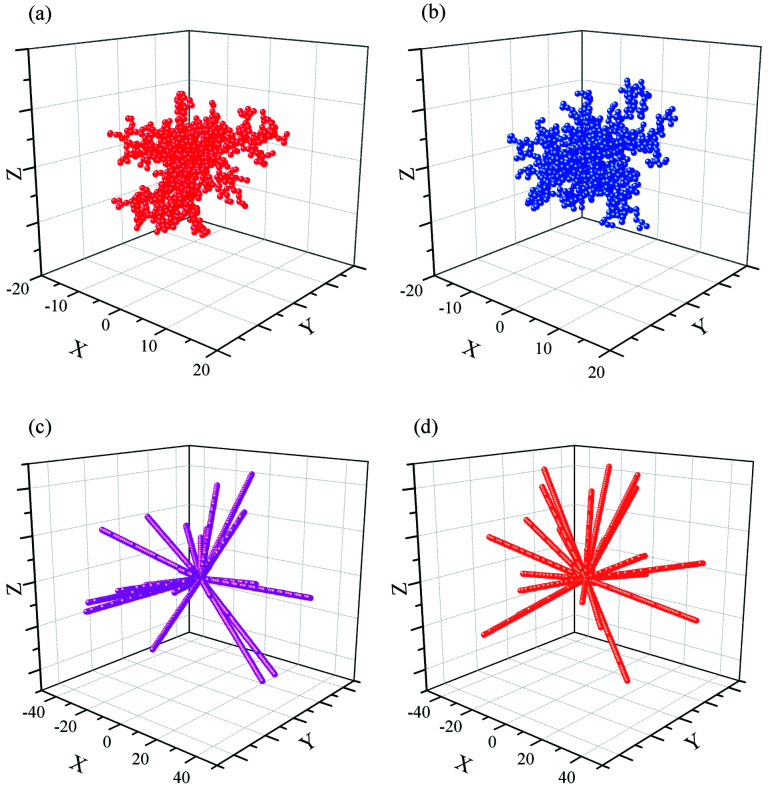
Random aggregate of 1000 particles on a 3D space with a basic point (0, 0, 0). *r*_max_ = 0.5. (a) Dist < 1; (b) Dist = 1; (c) Dist < 1, OA; (d) Dist = 1, OA.

Four typical clusters grown in 3D space with a sticking probability of 1.0 at nearest-neighbor sites, as shown in Fig. 3 and Fig. 5s.† The similar simulation of DLA in 2D plane is exhibited in Fig. 2b and 3s.† It is obviously a pine-needle like hierarchical structure is formed in the interaction between DLA and OA. Compared with the clusters (Fig. 3a and b) formed with DLA, the clusters (Fig. 3c and d) are more open and larger. Their inner regions are packed with fewer particles. Furthermore, the aggregation of particles becomes regular and quickly during their formation. All these are attributable to the synergy of Brownian motion and OA.

The fractal dimensionality (*D*) of the clusters was obtained by the radius of gyration method.^14,61,62^ The gyration radius (*R*_g_) is an average value of the proximity radius (the distance to the basic point) for particles. It can be calculated by eqn (4):4
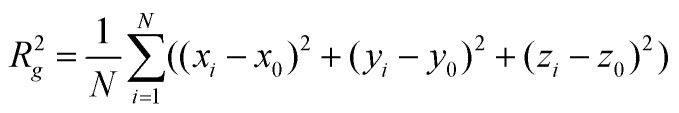
where *x*_*i*_, *y*_*i*_ and *z*_*i*_ are the coordinates of the particle, *x*_0_, *y*_0_ and *z*_0_ are the coordinates of the basic point (0,0, 0), *N* is the number of particles with the similar particle radius.

The number of particles in gyration radius (*N*_p_) scales with *R*_g_ as:

5 *N*_p_ ∼ *R*_g_^D^

**Fig. 4:**
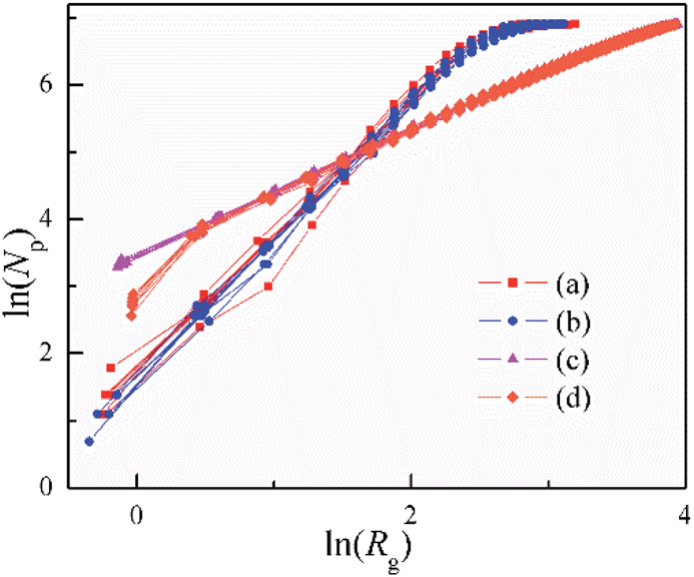
Dependence of the number of particles (*N*_p_) on gyration radius (*R*_g_) for the 3D clusters shown in Fig. 3.

The results of calculation are shown in Table 1. The 3D clusters of DLA in Fig. 3a and b) have *D* = 1.94 ∼ 2.09, and change little from 50% to 95% during the cluster formation. While in previous reports,^62–64^ the fractal dimensionality of the 3D DLA clusters with a sticking probability of 1.0 at nearest-neighbor sites are very close to *D* = 2.50. The 2D cluster of DLA in Fig. 2b and 3s† has *D* = 1.53∼1.62. This value is slightly smaller than the asymptotic fractal dimensionality of 1.71 found in diverse works.^10–14,58,63^ The 2D clusters with different restrictions of Brownian motion have very closed morphology and fractal dimensionality (Fig. 3s, 4s and Table 1s†), even with the increase of the max free path. However, the simulated values are always lower than the earlier DLA simulations with constrained movement distance and direction. This is because the random movement of particle make it easy to cross over the outer “arms” of the growing cluster, especially in 3D space. As a result, the present simulations without any restricted movement become closer to the reality DLA.

**Table 1:** The fractal dimensionality of the clusters shown in Fig. 2, 3 and 5s

Entry	Radius of gyration (*R*_g_)		Fractal dimensionality (*D*)		
	50%	95%	50%	75%	95%
(a)	9.1 ± 0.5	15.5 ± 2.5	2.02 ± 0.11	2.01 ± 0.15	1.94 ± 0.16
(b)	9.2 ± 0.4	16.0 ± 1.5	2.09 ± 0.11	2.05 ± 0.08	1.96 ± 0.07
(c)	19.5 ± 1.1	46.6 ± 1.2	0.94 ± 0.15	0.92 ± 0.08	0.89 ± 0.06
(d)	19.8 ± 1.3	46.5 ± 1.1	1.06 ± 0.08	1.00 ± 0.12	0.95 ± 0.11
Fig. 2b	91.5	158.5	1.61	1.62	1.59

The values of clusters with a sticking of Dist = 1 (Fig. 4d) show bigger fluctuation in the beginning than that with a sticking of variable distance (Fig. 4c). Nonetheless, they are very close and with the same structure. The sticking with fixed distance is introduced for the agglomeration of the rigid balls, as well as the formation of the inflexible pine-needle like nanostructure. Note that, the clusters formed with DLA and OA have *D* = 0.89 ∼ 1.06, and hold a good linear dependence for ln(*R*_g_) *versus* ln(*N*_p_) during their formation. Especially for the clusters with a sticking of Dist = 1, the fractal dimensionality of 0.95 ∼ 1.06 is quite close to Euclidean dimension of 1, and implies the radially from an origin point to three-dimensional space. This is in excellent agree with the pine-needle like structure of the clusters.

**Fig. 5:**
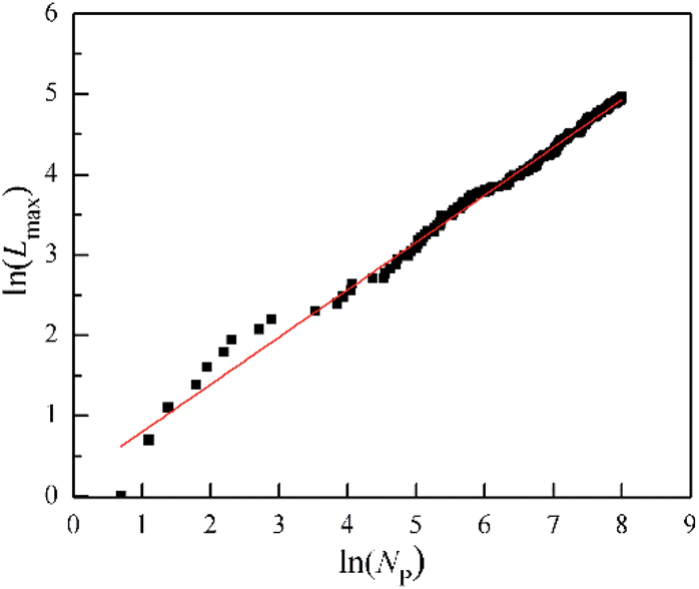
The change of the maximum length of needels (*L*_max_) with the number of particles (*N*_p_) for the DLA and OA cluster in the Fig. 6s.†

**Fig. 6:**
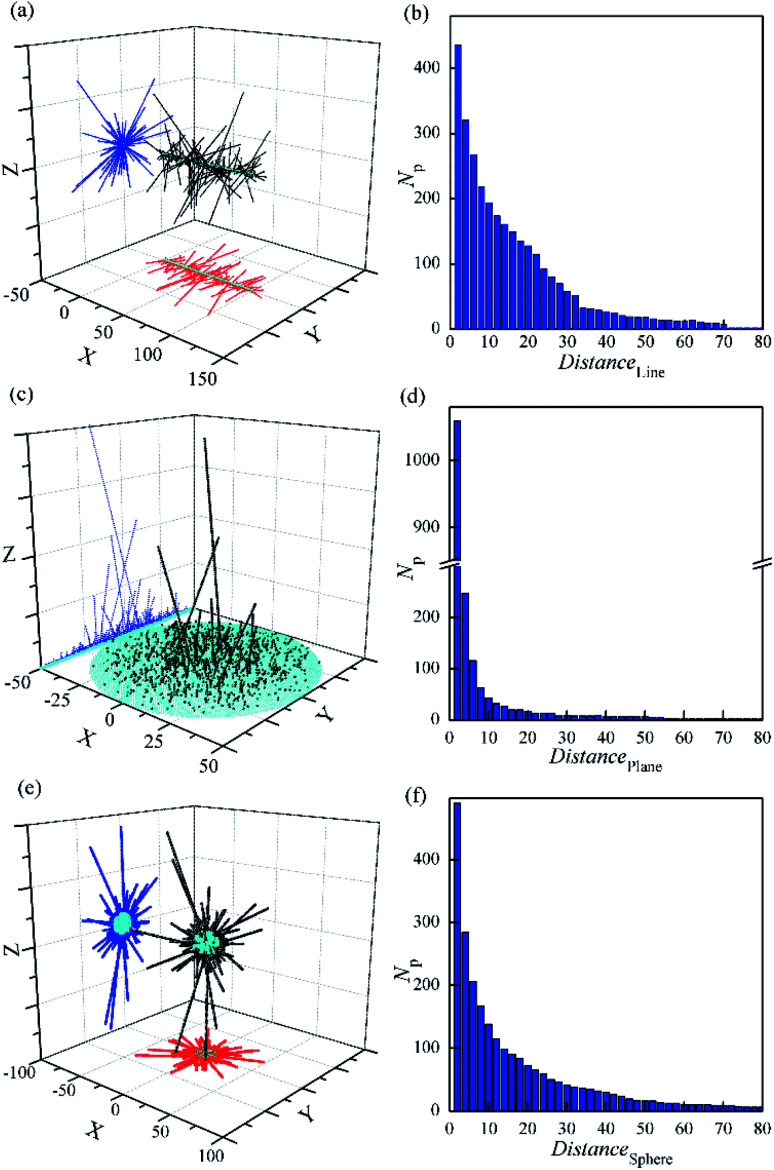
Random aggregate of 3000 particles by DLA and OA with a sticking of Dist = 1 based on line (a and b), plane (c and d) and sphere (e and f). *r*_max_ = 0.5.

For the growth process of the cluster of DLA and OA, Fig. 6s† presents the gradual formation from the inside out. The change of the maximum length of needels (*L*_max_) quantifies this process by the increased number of particles (*N*_p_) in Fig. 5. It is worth noting that the logarithms of *L*_max_ and *N*_p_ have a good linear relationship with R-Square 0.99. This indicates that the maximum length of needels is depends on the number of particles, and satisfies the following relation.

6 *L*_max_ = *N*_p_^b^

where *a* and *b* are constant (*a* = 1.2372, *b* = 0.5895 in Fig. 6s†). The more particles in the cluster, this relationship becomes more tightly over the whole range.

### 3.3 Random aggregate on a line, plane and sphere

**Fig. 7:**
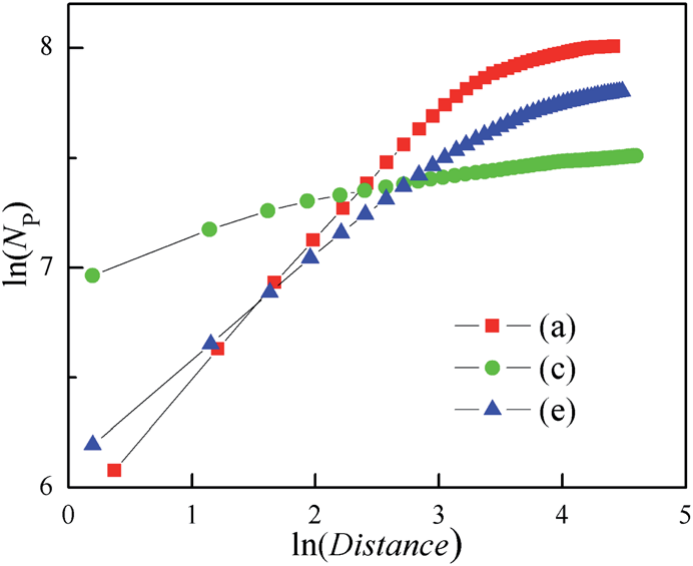
Dependence of *N*_Dis_ on Distance for the 3D cluster as shown in Fig. 5.

In order to match the actual pine-needle like structure, the random aggregate by DLA and OA on different bases were simulated, as shown in Fig. 6 and 7s.† It is obvious that only a few of the needles keep growing in the late formation. Especially based on plane in Fig. 6c, only one needle traps almost all the late particles. However, the realistic pine-needle like structure has no the very prominent needle,^49–52^ like the DLA and OA on a point. This is mainly because the particle density in real systems is so high that the growth rate becomes greater, rather than one by one.

The particles are more inclined to stick close to the centre of symmetry in Fig. 6a and c. This is in contrast with the previous simulation with only DLA on a line.^65^ These observations are in agreement with earlier DLA on a line without branching.^66^ In addition, the orientations of needles are almost perpendicular to the basis, despite the aggregation with random movement of particles. This is attributable to that the movement radius of particles (*r*_max_ = 0.5) is smaller than the sticking distance of 1.0. It is in accordance with the actual characteristic of the report.^49–52^

Furthermore, the distribution of particles was investigated. It should be noted that even though no surface with a basis of line (Fig. 6b), the number of particles with the same distance near the basis is still large and close to that based on the sphere (Fig. 5f). In contrast, that with a basis of plane (Fig. 6d) has only 2.2 times that based on the sphere, in spite of the area is 25 times that of the later. Moreover, the *N*_p_ value has the fastest decline with the increasing of the distance from the basis (Distance) in the three. It seems that the diffusion particles are difficult to cross the steric hindrance constructed by the outer needles on plane. This could be a quantitative understanding of how and why only a few needles grow during some preparations of pine-needle-like micro/nanostructures on plane.^51,52,67^ Because of relatively low steric hindrance on the line and sphere, the growth of needles is divergent to all around. And more needles stand out in the cluster compared with that on plane. This is similar to the aggregation with a basic point on the 3D space, and reveals the formation mechanism of some longer nano-needles on the line^49,50^ and sphere.^55,57,68,69^

A similar calculation to the radius of gyration method has been used to analysis the 3D clusters shown in Fig. 6. Here, the Distance was instead of gyration radius. The ln–ln plots of *N*p *vs.* Distance were shown in Fig. 7. This figure illustrates that Distance is related to *N*p as:

7 *N*p ∼ Distance^*β*^

This relation is similar with eqn (5), although the *β* is different from fractal dimension. The *β* values all decrease with the increasing of Distance, as well as the growth of the outer needles in the three clusters. In particular, the cluster with the basic plane has the minimum *β* only about 0.12. These imply that the *β* is able to evaluate the steric hindrance of needles.

## 4. Conclusions

In this paper, the combination model of DLA and OA was developed to describe the growth process of some real pine-needle like micro/nanostructures. Due to the introduction of OA, the cluster by DLA changes from random branches to regular needles. Moreover, the fractal dimensionality is very close to 1.0 during the whole growth process. With any bases, the formation of needles has always some steric hindrance from the outer needles. In particularly, only one needle grows up in the later period in the plane. It is hoped that the model could provide a quantitative understanding of the growth process of the aggregations with the pine-needle like structure and other similar structures.

## Author contributions

Zhijun Xia: conceptualization, modeling, software, analysis and investigation, data analysis, writing (original draft, review and editing).

## Conflicts of interest

There are no conflicts to declare.

## Supplementary Material

RA-012-D2RA03649E-s001
