# Supplementary material for: The growth simulation of pine-needle like structure with diffusion-limited aggregation and oriented attachment
Source: RSC Adv. 2022 Aug 15;12(35):22946–50. doi: 10.1039/d2ra03649e (PMC9377209; doi:10.1039/d2ra03649e)
Supplement: RA-012-D2RA03649E-s001 [file RA-012-D2RA03649E-s001.pdf]

Electronic Supplementary Material (ESI) for RSC Advances.
This journal is © The Royal Society of Chemistry 2022

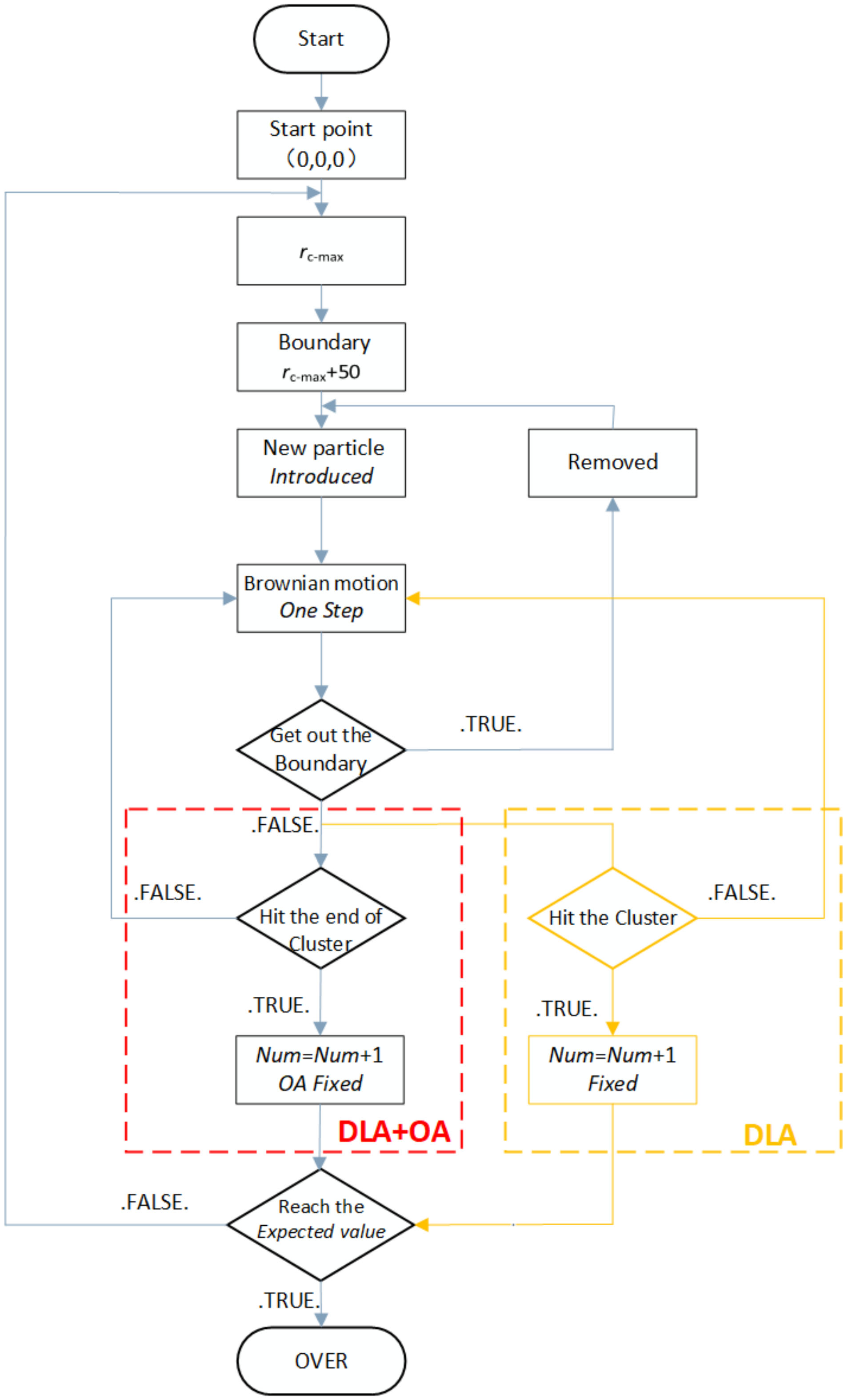

Fig. 1s The logic diagram of simulation process for DLA+OA and DLA. Where *Num* is the expected number of particles in the cluster.

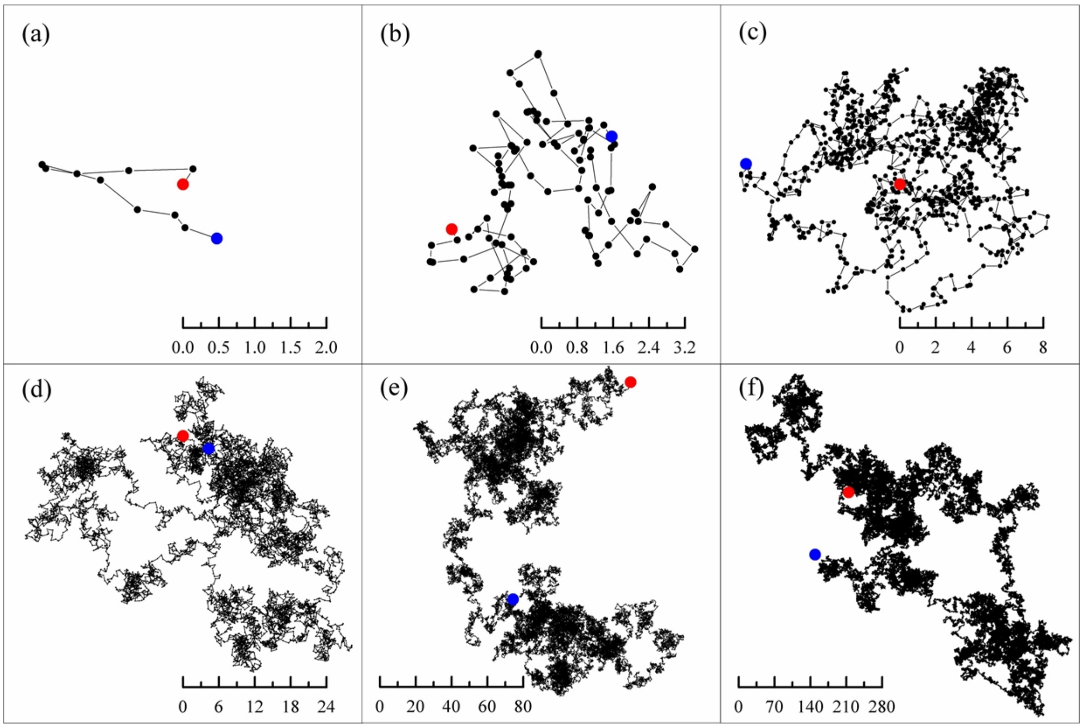

Fig. 2s Brownian motion of one particle in 2D plane with different steps. r_{max}=1. Starting point (0, 0,0). (a)10; (b)100; (c)1 000; (d)10 000; (e)100 000; (f)1 000 000. Red: Start; Blue: End.

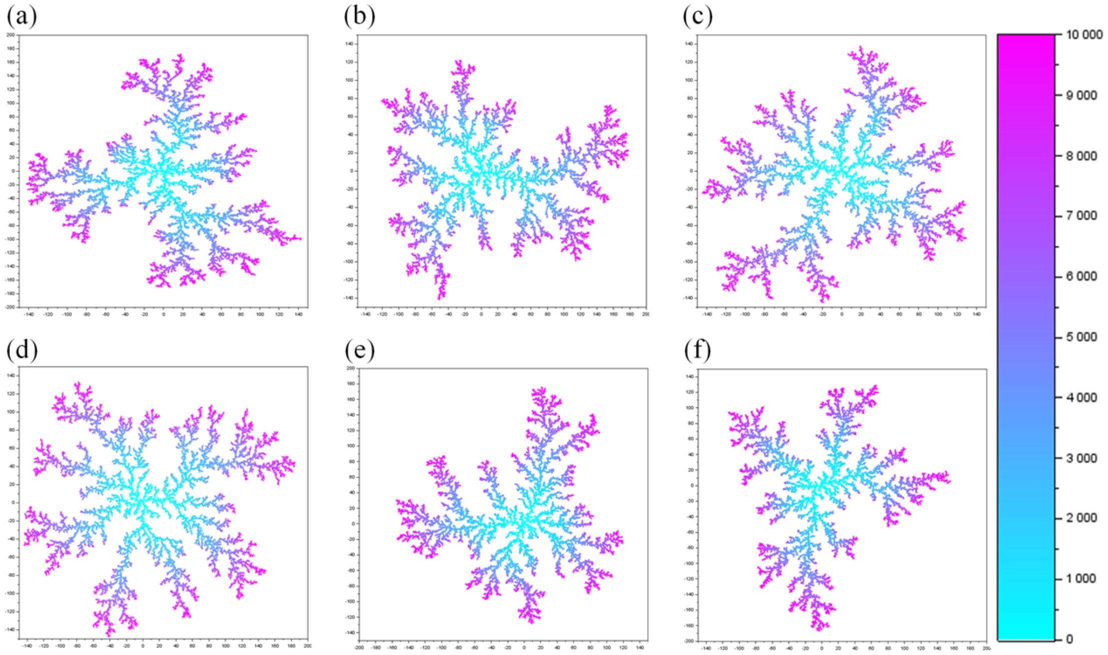

Fig. 3s Random aggregate of DLA with 10000 particles on a 2D plane with a basic point (0, 0). (a) r_{max}=0.5, Dist<1, r and θ randomly, ϕ=0; (b) r=0.5, Dist<1, θ randomly, ϕ=0; (c) r=0.5, Dist<1, θ= 0, 0.5π, π or 1.5π, ϕ=0; (d) r_{max}=0.25, Dist<1, r and θ randomly, ϕ=0; (e) r_{max}=0.75, Dist<1, r and θ randomly, ϕ=0; (f) r_{max}=0.99, Dist<1, r and θ randomly, ϕ=0.

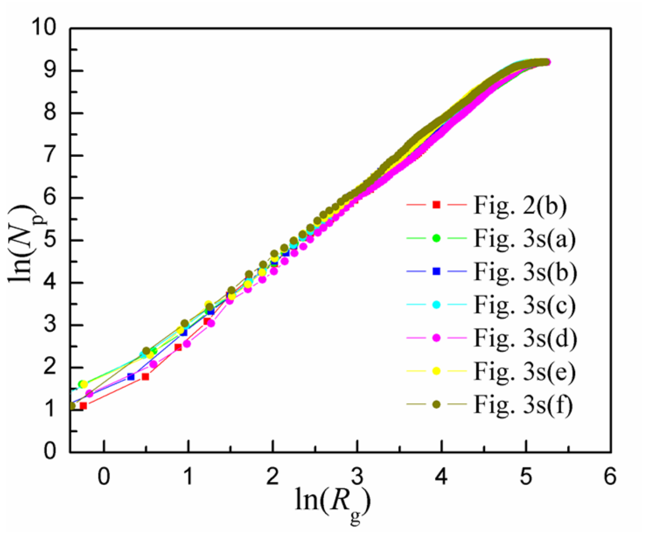

Fig. 4s The relationship of N_p and R_g for the 2D cluster as shown in Fig. 2(b) and Fig. 3s.

Table 1s The fractal dimensionality of the clusters shown in Fig. 3s.

Entry	Radius of gyration (R_g)		Fractal dimensionality (D)		
	50%	95%	50%	75%	95%
(a)	94.5	158.5	1.55	1.54	1.53
(b)	87.5	153.4	1.59	1.59	1.56
(c)	84.5	142.4	1.59	1.60	1.58
(d)	94.5	153.5	1.61	1.62	1.61
(e)	81.5	145.5	1.60	1.60	1.57
(f)	83.5	149.5	1.59	1.59	1.55

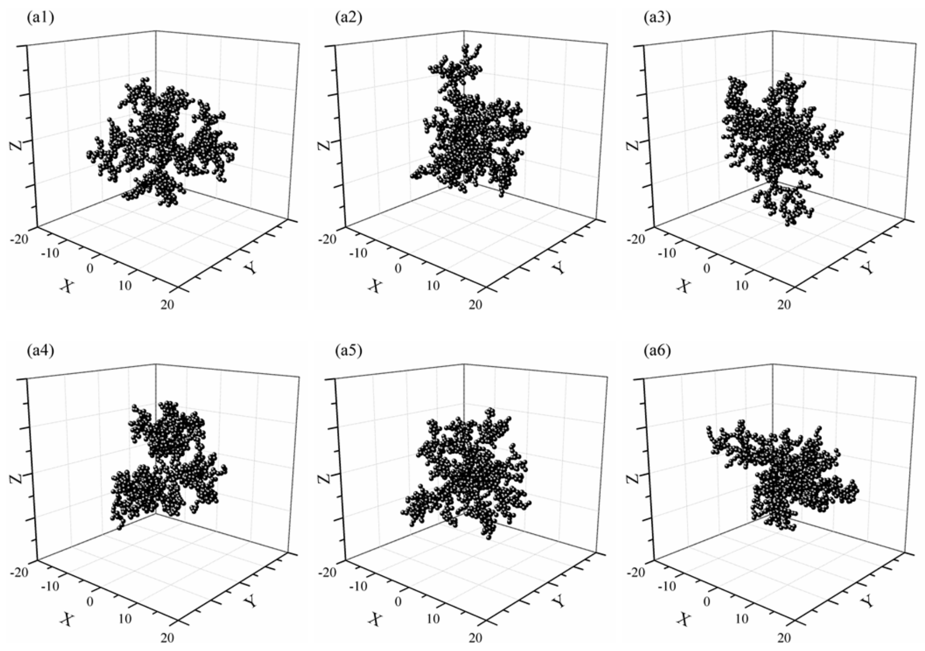

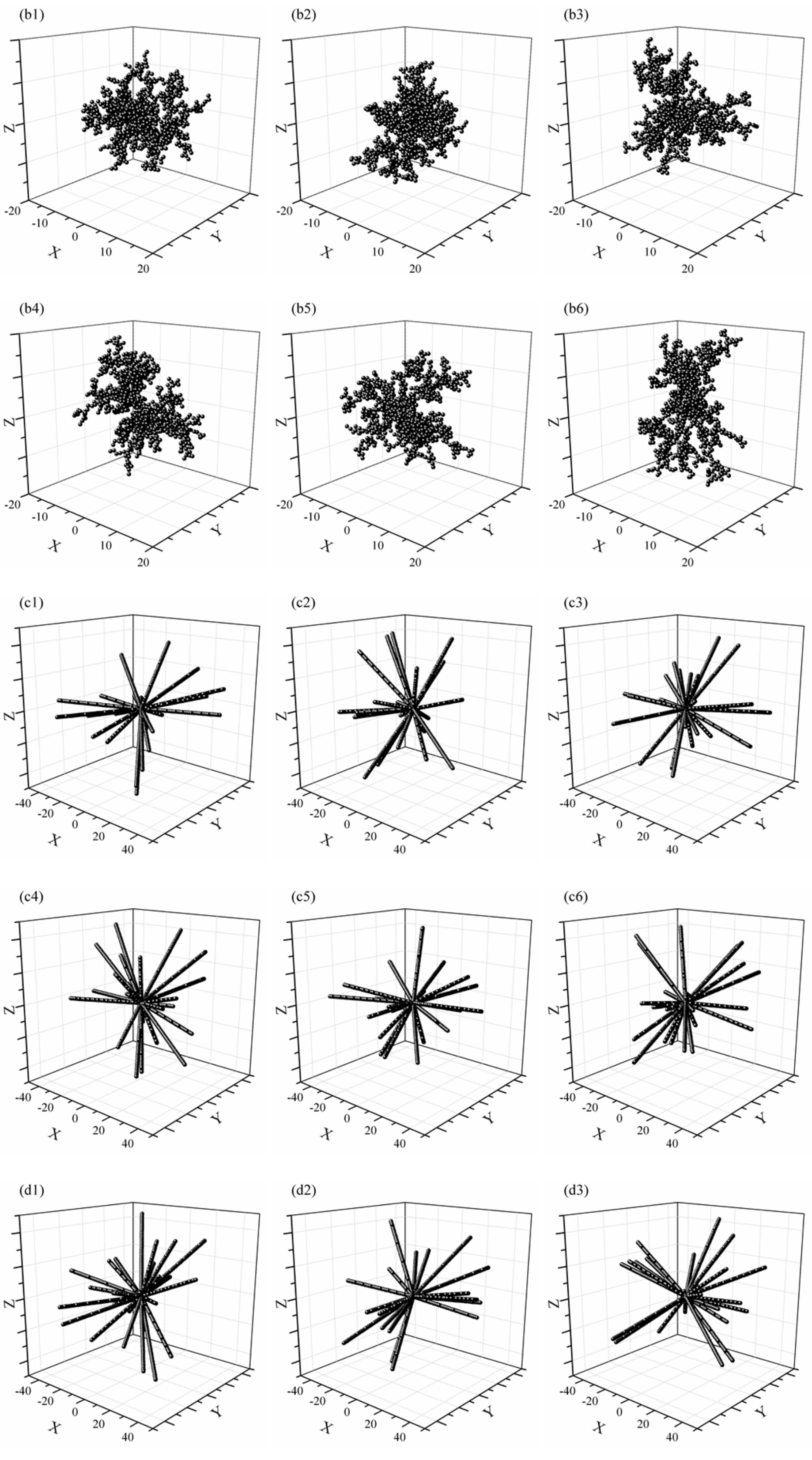
(b1)
(b2)
(b3)
(b4)
(b5)
(b6)
(c1)
(c2)
(c3)
(c4)
(c5)
(c6)
(d1)
(d2)
(d3)
X
Y
Z
-20
-10
0
10
20
-40
-20
0
20
40

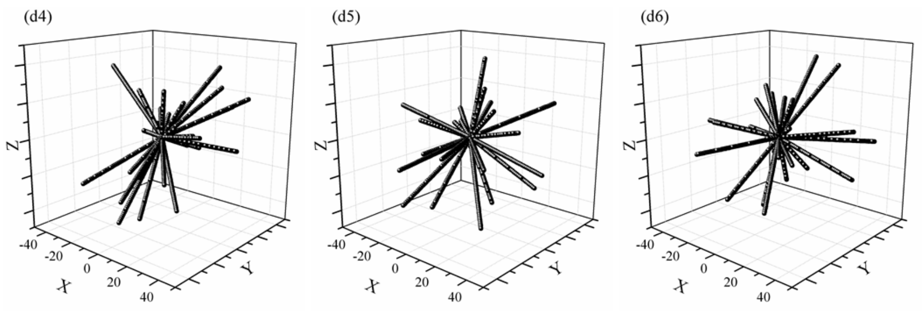

Fig. 5s The replication of random aggregate with 1000 particles as shown in Fig 2. Basic point (0, 0, 0), r_{max}=0.5. (a1~a6) *Dist*<1; (b1~b6) *Dist*=1; (c1~c6) *Dist*<1, OA; (d1~d6) *Dist*=1, OA.

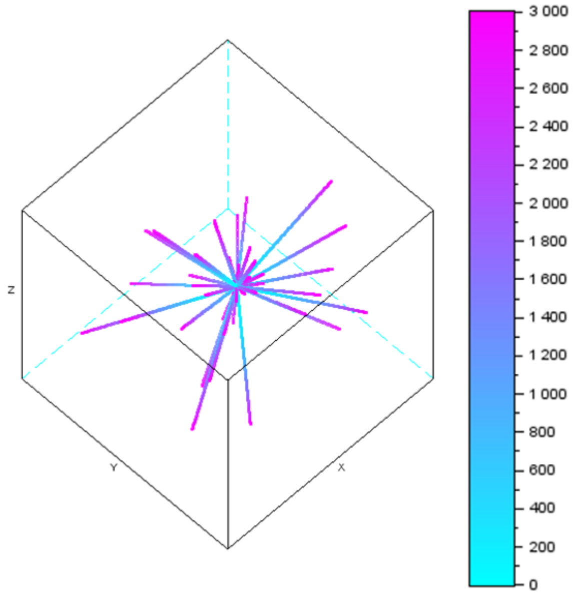

Fig. 6s The random aggregate process of DLA and OA with 3000 particles. Basic point (0, 0, 0), r_{max}=0.5, *Dist*=1.

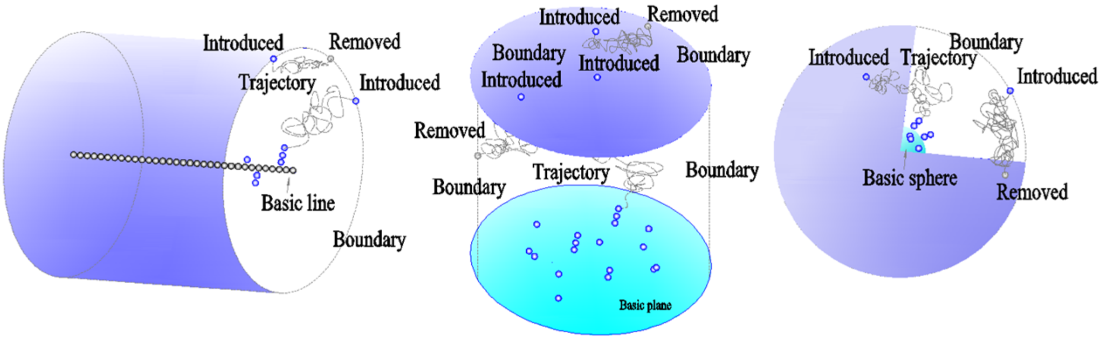

（a） （b） （c）

Fig. 7s The simulation models for random aggregate with DLA and OA based on line (a), plane (b) and sphere (c).
